# EEG and transcranial dopplerography of middle cerebral artery in patiens with primary headaches

**DOI:** 10.1186/1129-2377-14-S1-P134

**Published:** 2013-02-21

**Authors:** UG Askhonov, GH Askhanov

**Affiliations:** 1 Neurological clinic "Nevromed-Servis", Uzbekistan

## 

The objective of this study was to evaluate relationships between EEG and TCD changes of middle cerebral artery (MCA) in patients with primary headaches. Seventy seven patients with primary headaches(age=24,2±7.0) and 15 normal subjects were assessed using ICHD-2, neurological evaluation, visual analogue scale (1-10 points), transcranial dopplerography (TCD) and an EEG analysis of absolute and relative band amplitude at rest. Three groups were compared:30 patients with Tension headaches(TH),32 patients with migraine(MG),15 patients with cluster headache(CLH)and the control group. Were used dominant frequency, amplitude, index of waves, correlation analysis of EEG, standardized low resolution brain electromagnetic tomography (sLoreta) and maximum, minimum, average frequency, RI, PI by TCD of middle cerebral artery (MCA). The EEG showed significant differences between the control group and patients with TH,MG and CLH.Oscillation of delta in left frontal lobe (Brodmann area 11)in patients with TH, in the right frontal lobe in MG patients and bilateral in CH patient. The maximal density of theta in the left occipital lobe(Br.area 18) in TH patient, in right occipital lobe in MG and bilateral in CLH patients. In occipital lobe in patients with TH, MG and in frontal lobe in the patients with CH was find the maximal density of Alpha 1 waves, as well as the alpha 2 in occipital lobe in all patients. Maximal density of beta 1 was registered in the right occipital lobe in TH,MG patients and in frontal lobe in CLH patients. Gamma waves were registered in frontal lobe in all patients and in control group (F(1,56)=18,2.p<0.001). Abnormalities on the EEG were essentially associated with the occurrence of increasing of the frequency and dominate in patients with MG and CH. Were found relationship between dominant frequency of EEG and TCD parameters (Vmax, Ri,Pi) p>0,01. Were determined that TCD parameters such as RI, PI decreased in MG,CLH patients (p<0.001) This study suggested EEG and TCD of MCA as a possible physiological tool in the assessment of pathogenesis of headaches.

## Medical equipments

EEG is a product of the company "Mitsar", Russia TCD is a product of company "Spectromed", Russia

